# Histone deacetylase inhibitors enhance the amoebicidal activity of amphotericin B against *Naegleria fowleri*

**DOI:** 10.1016/j.ijpddr.2026.100664

**Published:** 2026-08-03

**Authors:** Hae-Ahm Lee, Hye-Jeong Jo, Ji-Youn Hong, Fu-Shi Quan, Eun-Kyung Moon

**Affiliations:** a Department of Medical Zoology, Kyung Hee University College of Medicine, Seoul, Republic of Korea; b Department of Biomedical Science, Graduate School, Kyung Hee University, Seoul, Republic of Korea; c Department of Periodontology, Kyung Hee University College of Dentistry, Periodontal-Implant Clinical Research Institute, Kyung Hee University Dental Hospital, Seoul, Republic of Korea

**Keywords:** *N. fowleri*, HDAC inhibitor, Amphotericin B, PAM

## Abstract

*Naegleria fowleri* is a free-living amoeba that causes a rare but almost always fatal brain infection known as primary amoebic meningoencephalitis (PAM). Although amphotericin B (AmB) is considered the primary treatment for PAM, it is often recommended in combination with several other drugs due to side effects associated with high concentrations. In this study, we investigated whether histone deacetylase inhibitors (HDACis) enhance the amoebicidal activity of AmB against *N. fowleri*. Anti-proliferative and cytotoxic activities were assessed using the cell counting kit and lactate dehydrogenase assays, respectively, and apoptosis induction was evaluated by flow cytometry. Class-specific HDACis, including class I inhibitors MS275 and MGCD0103 and a class II inhibitor MC1568, showed minimal effects on trophozoite viability. In contrast, the pan-HDACis SAHA and JNJ significantly reduced trophozoite viability in a dose- and time-dependent manner. Furthermore, 10 μM SAHA and JNJ significantly reduced the encystation ratio of *N. fowleri*. Combined treatment with low doses of HDACi (1 μM) and AmB (0.5 μM) produced stronger amoebicidal and pro-apoptotic effects than either agent alone. These combination treatments also exhibited low cytopathic effects on human brain tumor cells. These findings suggest that SAHA and JNJ, particularly in combination with AmB, hold promise as therapeutic candidates against *N. fowleri* infection while potentially mitigating the severe side effects associated with AmB.

## Highlights

• SAHA and JNJ reduced *N. fowleri* trophozoite viability in a dose-dependent manner.
• SAHA and JNJ induced cytotoxicity and apoptosis in *N. fowleri* trophozoites.
• SAHA and JNJ almost markedly inhibited cyst formation in *N. fowleri.*
• SAHA and JNJ enhanced the anti-amoebic activity of amphotericin B.
• SAHA and JNJ showed minimal cytotoxicity in the human cell line.

## Graphical abstract

**Figure:**
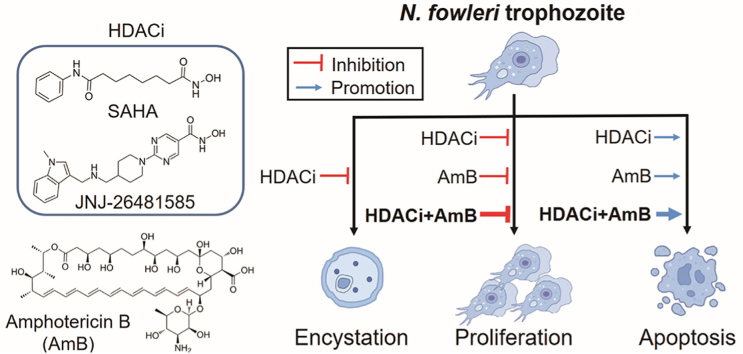

## 1 Introduction

*Naegleria fowleri*, commonly known as the "brain-eating amoeba," is a free-living, thermophilic microorganism prevalent in warm freshwater environments such as lakes, rivers, ponds, hot springs, and moist soil, particularly at temperatures between 25°C and 46°C (Leal Dos Santos et al., 2022). This amoeba is the causative agent of primary amoebic meningoencephalitis (PAM), a rare but severely life-threatening infection that affects the central nervous system (CNS). PAM typically occurs when contaminated water enters the nasal passages, allowing the amoeba to migrate to the brain via the olfactory nerve (Chen and Moseman, 2022). Upon reaching the brain parenchyma, *N. fowleri* trophozoites incite substantial inflammation, leading to the destruction of host brain tissue and subsequent CNS damage (Visvesvara et al., 2007). Factors such as climate change have expanded the regions and periods where *N. fowleri* can survive, resulting in a gradual increase in infection risk. Since 2000, there has been a rise in reported PAM cases (Maciver et al., 2020). The actual number of infections is likely underestimated due to inadequate surveillance and diagnostic challenges. Since the discovery of *N. fowleri*, annual reports have ranged from 0 to 8 cases, with a total of 488 PAM cases documented worldwide by 2023 (Alanazi et al., 2025). Despite its rarity, the high mortality rate associated with PAM, often exceeding 97%, underscores the critical need for effective therapeutic interventions (Guemez and Garcia, 2021).

To date, 11 individuals have survived *N. fowleri* infection following treatment, all of whom received AmB, either alone or in combination with other drugs such as azithromycin, miltefosine, rifampin, and dexamethasone (Burqi et al., 2024), administered intravenously or intrathecally. Accordingly, AmB remains the mainstay of treatment for PAM because of its amoebicidal activity against *N. fowleri* (Vargas-Zepeda et al., 2005; Guemez and Garcia, 2021; Alanazi et al., 2025). However, the major limitation of AmB in PAM is its limited efficacy in controlling this rapidly progressive and often fatal infection. In addition, although its clinical use is associated with severe adverse effects, particularly nephrotoxicity, which may lead to renal impairment and electrolyte disturbances (Laniado-Laborin and Cabrales-Vargas, 2009), these toxicities are secondary to the more fundamental challenge of inadequate disease control. Together, these limitations underscore the need for alternative or adjunctive therapeutic strategies to improve treatment outcomes in PAM (Soltow and Brenner, 2007).

Recently, histone deacetylase inhibitors (HDACis) have emerged as a promising class of compounds with potential applications in treating various diseases, including neurological, cardiovascular, kidney, inflammatory, and autoimmune disease, beyond their initial use in oncology (Zhou et al., 2024). By modifying chromatin structure and influencing gene expression, HDACis can induce cell cycle arrest, apoptosis, and differentiation in target cells (Xu et al., 2007). This mechanism of action suggests that HDACis could possess broad therapeutic potential, including activity against protozoan parasites. Supporting this rationale, the antiparasitic effects of HDACis have been reported, including their parasiticidal effects on malaria (Giannini et al., 2015), schistosomiasis (Guidi et al., 2018), trypanosomiasis (Kelly et al., 2012), toxoplasmosis (Strobl et al., 2007), leishmaniasis (de Souza et al., 2025), and trichomoniasis (Seifert-Gorzycki et al., 2025). Most recently, a study suggesting that HDACis inhibit the proliferation and encystation of *Acanthamoeba*, a type of free-living amoeba, indicating their potential as a therapeutic agent for treating amoebic keratitis (Chu et al., 2022). Furthermore, in high-throughput screening studies of free-living amoebae, the HDAC inhibitor panobinostat emerged as a potential therapeutic candidate against *N. fowleri* (Rice et al., 2020), further supporting the rationale for evaluating HDACis in this pathogen.

Building on previous research, this study sought to validate the amoebicidal activity of HDACis against *N. fowleri* using a broad panel of compounds, including both pan-HDACis and class-specific HDACis. In addition to evaluating the individual effects of these inhibitors, we investigated their combined administration with AmB to determine whether HDACis could potentiate or synergize with the current standard therapy. The study aimed to identify HDACis with efficacy against *N. fowleri* and to assess their effects - both alone and in combination with AmB - on the viability, encystation, cytotoxicity, and apoptosis of *N. fowleri*.

## 2 Materials and methods

### 2.1 Chemicals

Water-soluble amphotericin B (AmB) was purchased from Sigma Aldrich (St. Louis, MO, USA). The pan-HDACis suberoylanilide hydroxamic acid (SAHA) (Sigma Aldrich, St. Louis, MO, USA) and JNJ-26481585 (JNJ) (Tocris bioscience, Bristol, UK), along with the class I HDACis MS275 and MGCD0103, and the class II HDACi MC1568 (all from Cayman chemical, Ann Arbor, MI, USA) were prepared as 10 mM stock solutions in dimethyl sulfoxide (DMSO) and stored at −20°C until use. Cell counting kit (CCK) was purchased from Dojindo (Kumamoto, Japan). EzWay Annexin V-FITC Apoptosis Detection Kit was purchased from LABISKOMA (Seoul, Korea).

### 2.2 Cell culture

*Naegleria fowleri* (ATCC 30894) was obtained from the American Type Culture Collection (Manassas, VA, USA). *N. fowleri* were cultured axenically in Nelson's medium (MgSO_4_∙7H_2_O 4 mg, CaCl_2_∙2H_2_O 4 mg, Na_2_HPO_4_ 142 mg, KH_2_PO_4_ 136 mg, NaCl 12 mg, liver infusion 1.7 g, glucose 1.7 g and distilled water to make up 1 L) containing 10% fetal bovine serum (FBS) at 37°C. SNU-1118 cells were purchased from the Korean Cell Line Bank (Seoul, Korea) and cultured in RPMI 1640 medium (WelGENE, Daegu, Korea) with 10% FBS at 37°C in a 5% CO_2_ incubator.

### 2.3 Encystation

The cyst formation of *N. fowleri* was induced using MYAS medium, which consists of malt extract (0.1 g), yeast extract (0.1 g), and 1 L of amoebae saline (containing NaCl 120 mg, MgSO_4_∙7H_2_O 4 mg, CaCl_2_∙2H_2_O 4 mg, Na_2_HPO_4_ 142 mg, KH_2_PO_4_ 136 mg, and distilled water to make up 1 L) at 37°C. *N. fowleri* trophozoites were seeded at a density of 1 × 10^4^ cells per well in 96-well plates containing 200 μl of MYAS medium, and treated with or without 10 μM of HDAC inhibitors (HDACis). The plates were incubated at 37°C for 7 days. After incubation, the transformation from trophozoite to cyst was observed using light microscopy (Leica DMi8, Wetzlar, Germany). Encystation efficiency was assessed by counting the number of cysts under a light microscope before and after treating the cells with 0.5% sodium dodecyl sulfate (SDS).

### 2.4 Viability test

To investigate the effect of HDACis and AmB on the viability of *N. fowleri*, a water-soluble formazan-forming CCK (Cell counting kit) assay was performed. *N. fowleri* were seeded in a 96-well plate at a density of 1 × 10^4^ cells per well and incubated overnight to allow for cell attachment. HDACis were administered at concentrations ranging from 0 to 10 μM, followed by incubation for 24 or 48 h. Following treatment, 20 μl of CCK reagent was added to each well, and the plate was incubated at 37°C for 4 h. After incubation, optical density was measured at 490 nm using a microplate reader. All assays were performed in triplicate. IC_50_ values were calculated from the dose-response data by nonlinear regression analysis using GraphPad Prism 8 (Dotmatics, San Diego, CA, USA).

### 2.5 LDH assay

Cytotoxicity induced by HDACis in *N. fowleri* was assessed using the Quanti-LDH™ PLUS Cytotoxicity Assay Kit (BIOMAX, Guri, Korea). *N. fowleri* was seeded in a 96-well plate at a density of 1 × 10^4^ cells in 100 μl of culture media containing 2.5% of heat-inactivated FBS and incubated overnight to allow for cell attachment. After treatment with HDACis, 100 μl of substrate solution was added to each well, and the plate was incubated at room temperature for 30 min. Absorbance was measured at 490 nm using a microplate reader. Wells containing *N. fowleri* completely lysed with lysis buffer were used as the positive control. The *in vitro* cytotoxicity was calculated as follows: cytotoxicity (%) = [(sample release − spontaneous release)/(maximum release − spontaneous release)] × 100. All assays were performed in triplicate.

### 2.6 Flow cytometry analysis

Apoptosis-like cell death in *N. fowleri* induced by AmB, HDACis, or their combination was analyzed using EzWay Annexin V-FITC Apoptosis Detection Kit. *N. fowleri* was seeded in a T-25 culture flask at a density of 5 × 10^5^ cells in 5 ml of culture media and incubated overnight to allow for cell attachment. After the administration of AmB, HDACis or their combination for 24 or 48 h, the cells were detached from the flask by brief incubation on ice and gentle shaking. The cells were harvested by centrifugation at 1,000 g for 5 min and washed with cold PBS. The washed cells were resuspended in 0.5 ml of binding buffer. The cells were stained with FITC-conjugated Annexin V (0.5 μg/ml) for 30 min at room temperature in the dark. The supernatant was removed after centrifugation at 1,000 g for 5 min. Cells were resuspended in 0.5 ml of binding buffer containing propidium iodide (1 μg/ml). Flow cytometry analysis was performed using BD Accuri C6 (BD Biosciences, San-Jose, CA, USA).

### 2.7 Mitochondrial membrane potential analysis

Mitochondrial membrane depolarization was analyzed using a mitochondrial membrane potential kit (Merck, New Jersey, USA). A total of 1 × 10^5^ *N. fowleri* cells were seeded into 12-well plates and incubated for 24 h. The cells were then treated with AmB (0.25 μM), SAHA (2.5 μM), and JNJ (2.5 μM), either alone or in combination, and further incubated for 24 h. JC-10 dye was added, and the cells were incubated in the dark for 1 h. Fluorescence images were then captured using a fluorescence microscope (Leica DMi8, Wetzlar, Germany).

### 2.8 Statistical analysis

Data are presented as mean ± SD from three independent experiments. Student's *t*-tests and ANOVA were performed using GraphPad Prism version 8. Statistical significance between the means of groups was indicated using asterisks, with P values less than 0.05 considered statistically significant (*P < 0.05, and **P < 0.01).

## 3 Results

### 3.1 Pan-HDACis treatment inhibited the viability of *N. fowleri* trophozoites

**Fig. 1:**
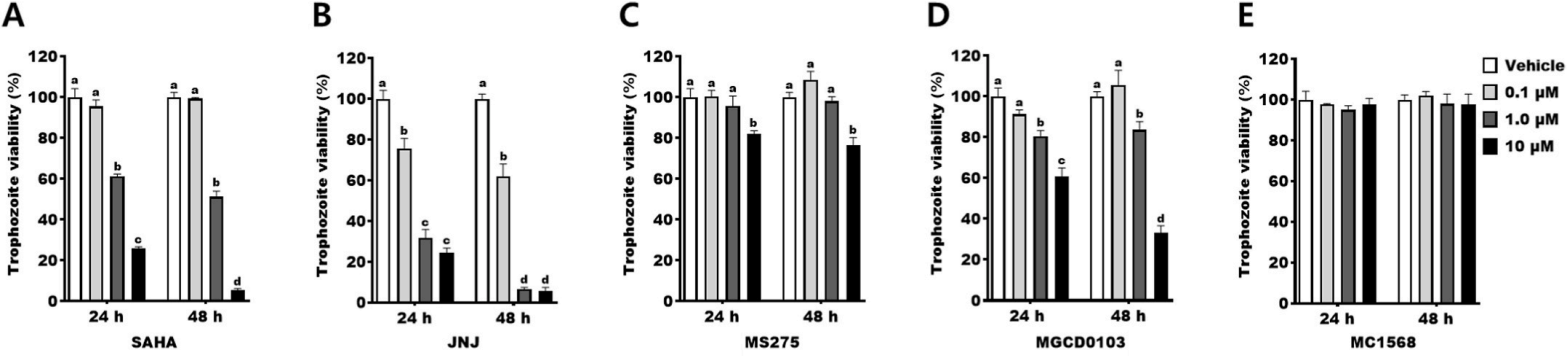
Pan-HDACis demonstrated significant amoebicidal activity against *N. fowleri* trophozoites. The CCK assay was used to evaluate the viability of *N. fowleri* trophozoites following 24 and 48 h of treatment with SAHA (A), JNJ (B), MS275 (C), MGCD0103 (D), or MC1568 (E) at the indicated concentrations. Treatment with SAHA and JNJ resulted in a statistically significant reduction in trophozoite viability compared to the vehicle control. Data represent the mean ± S.D. of three independent experiments. Statistical significance was analyzed by two-way ANOVA followed by Sidak's multiple-comparisons test. Different characters indicate statistically significant differences (*p* < 0.05).

To evaluate the amoebicidal effects of HDACis on *N. fowleri*, five different HDACis were administered to the trophozoite of *N. fowleri* (Fig. 1). SAHA and JNJ are classified as pan-HDACis, whereas MS275 and MGCD0103 are class I HDACis, and MC1568 is a class II HDACi. Treatment with 0.1 μM SAHA for either 24 or 48 h resulted in negligible changes in the viability of *N. fowleri*. However, treatment with 1 μM and 10 μM SAHA for 24 h reduced the viability of *N. fowleri* to 61.3% and 35.7% of the control, respectively, and 48 h treatment significantly reduced it to 51.3% and 5.3% respectively. (Fig. 1A). *N. fowleri* showed greater sensitivity to JNJ than to SAHA, with cell viability of 75.7% and 62.1% after 24 and 48 h of treatment at 0.1 μM JNJ, respectively. When treated with 1 and 10 μM JNJ for 24 h, the viability of *N. fowleri* decreased to 31.8% and 24.5%, respectively, and after 48 h of treatment, it was markedly reduced to 6.7% and 5.7% (Fig. 1B). The class I HDAC inhibitor MS275 showed negligible change at low concentrations (∼1 μM), and when treated at 10 μM for 24 and 48 h, the viability of *N. fowleri* decreased to 81.9% and 76.3%, respectively (Fig. 1C). Another class I HDAC inhibitor MGCD0103 showed a stronger inhibitory effect on *N. fowleri* viability than MS275. At 0.1 μM, it did not affect viability, whereas treatment with 1 and 10 μM for 24 h reduced viability to 80.5% and 60.8%, respectively. After 48 h of treatment, viability decreased to 83.7% and 32.9%, respectively (Fig. 1D). The class II HDAC inhibitor MC1568 showed no effect on the viability of *N. fowleri* even after 24 or 48 h of treatment at concentrations up to 10 μM (Fig. 1E).

### 3.2 SAHA and JNJ induced cytotoxicity in *N. fowleri*

**Fig. 2:**
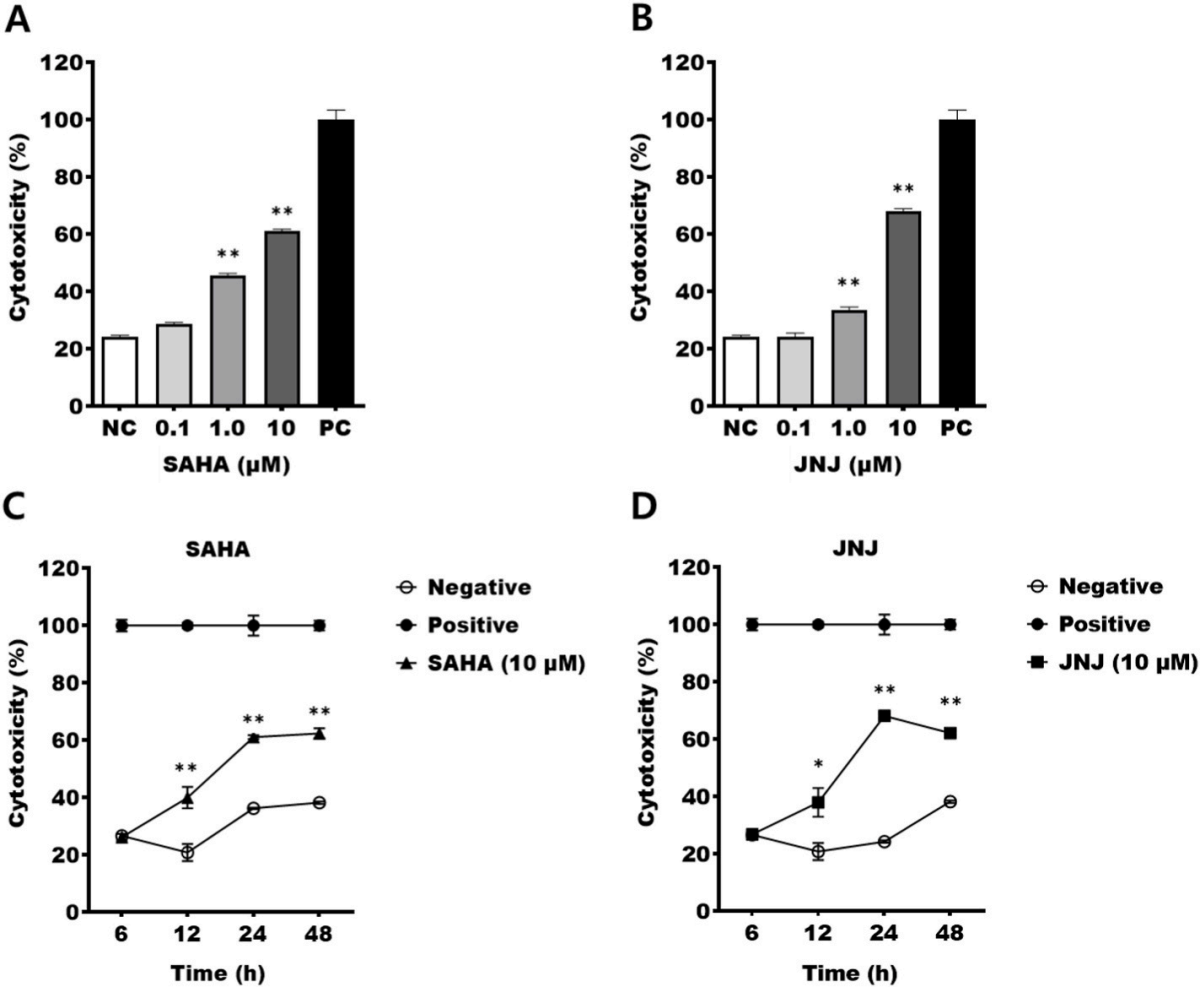
Cytotoxicity analysis of HDACis on *N. fowleri* trophozoites. The LDH assay was conducted to assess the cytotoxic effects of HDACis on *N. fowleri* trophozoites. A and B showed a significant increase in cytotoxicity with SAHA and JNJ in a dose-dependent manner for 24 h, respectively. C and D demonstrated a significant enhancement in cytotoxicity with 10 μM of SAHA and JNJ in a time-dependent manner, respectively. Data are presented as the mean ± S.D. from three independent experiments. Statistical differences were determined using a Student's *t*-test for A and B, or repeated measures ANOVA for C and D (*P < 0.05, and **P < 0.01).

HDACi-induced cytotoxicity in *N. fowleri* was demonstrated by an LDH assay. Treatment with SAHA and JNJ increased cytotoxicity in a dose- and time-dependent manner (Fig. 2). The cytotoxicity induced by 0.1 μM SAHA for 24 h was negligible compared to the vehicle. However, treatment with 1 μM SAHA increased cytotoxicity to approximately 21.4%, while 10 μM SAHA increased it to approximately 36.8%, compared with the vehicle control (Fig. 2A). JNJ also exhibited a dose-dependent enhancement of cytotoxicity in *N. fowleri*. Compared to the vehicle control, treatment with 1 μM JNJ resulted in a roughly 10.2% increase in cytotoxicity, while 10 μM JNJ led to an approximate 43.8% increase (Fig. 2B). To investigate whether extending HDACi treatment beyond 24 h would further increase cytotoxicity, *N. fowleri* was treated with HDACi for 48 h. However, this prolonged treatment did not result in an additional increase in cytotoxicity. To evaluate time-dependent cytotoxicity, the short-term effects of HDACis on *N. fowleri* were investigated at time points earlier than 24 h. Both SAHA and JNJ exhibited similar cytotoxic effects, with insignificant changes observed at 6 h. However, there was a significant increase in cytotoxicity, with approximately 19.1% and 17.1% at 12 h, and 24.8% and 43.8% at 24 h for SAHA (Fig. 2C) and JNJ (Fig. 2D), respectively.

### 3.3 SAHA and JNJ treatment increased apoptosis-like cell death of *N. fowleri*

**Fig. 3:**
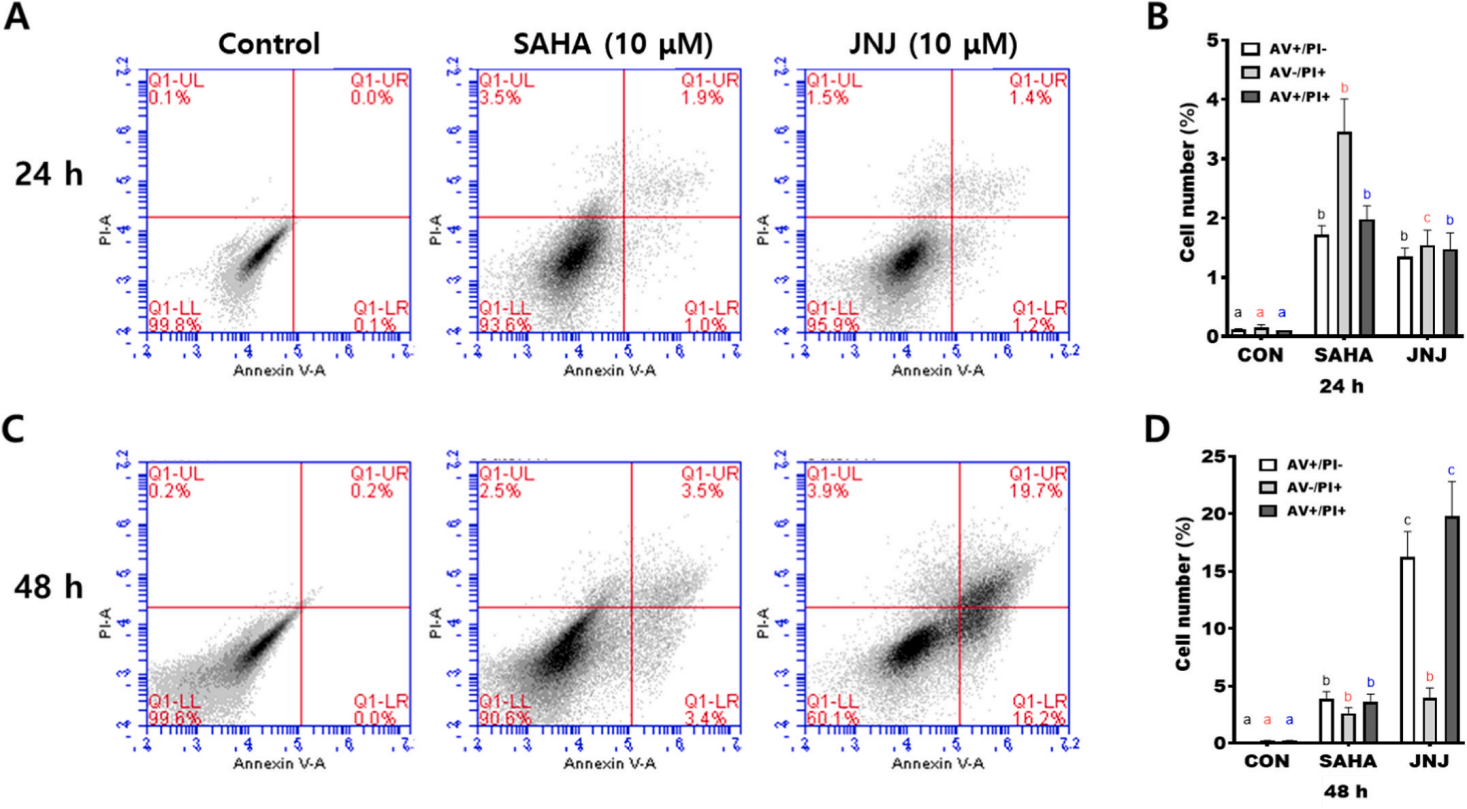
HDACis-induced apoptosis-like cell death in *N. fowleri*. Apoptosis-like cell death in *N. fowleri* induced by HDACis treatment was analyzed using flow cytometry following Annexin V and PI staining. A and B show that treatment with 10 μM SAHA or JNJ resulted in a slight increase in early and late apoptosis-like cell death in *N. fowleri*, whereas C and D show that treatment with 10 μM SAHA or JNJ for 48 h led to a greater increase in apoptosis-like cell death. The bar graphs represent the mean ± S.D. from three independent experiments. Statistical significance was analyzed using one-way ANOVA followed by Dunnett's post hoc test. Different characters indicate statistically significant differences at *p* < 0.05.

The apoptosis-like cell death of *N. fowleri* induced by HDACis was analyzed using flow cytometry analysis after staining with annexin V and PI (Fig. 3). Administration of 10 μM SAHA and JNJ led to an increase in apoptotic-like cells in *N. fowleri*, although the effects varied. Following 24 h of SAHA treatment, approximately 2.9% of *N. fowleri* cells were apoptotic-like, comprising 1.0% early apoptotic-like and 1.9% late apoptotic-like cells. After 48 h of SAHA treatment, apoptosis-like cell death in *N. fowleri* further increased, with early apoptosis-like cell death at 3.4% and late apoptosis-like cell death at 3.5%. (Fig. 3A and B). Apoptosis-like cell death induced by 10 μM JNJ after 24 h was observed at levels similar to those of SAHA, with early apoptosis-like cell death at 1.2% and apoptosis-like cell death at 1.4%. A dramatic elevation was observed after 48 h of treatment, with early apoptosis-like cell death increasing to 16.2% and late apoptosis-like cell death to 19.7%. (Fig. 3C and D).

### 3.4 SAHA and JNJ inhibited encystation of *N. fowleri*

**Fig. 4:**
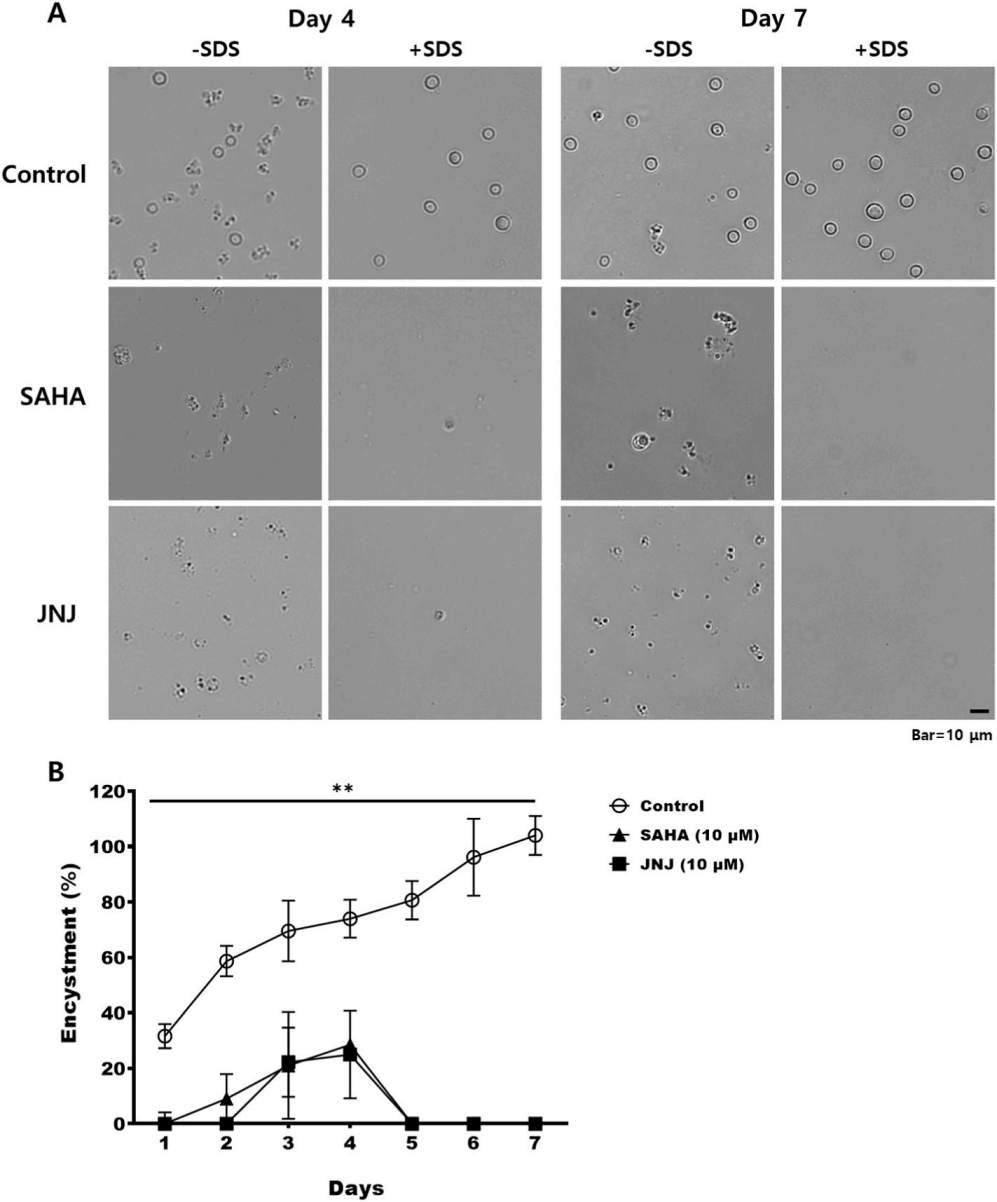
Effect of HDACis on the encystation of *N. fowleri*. The number of mature cysts induced by MYAS medium was counted under a light microscope before and after incubation with 0.5% SDS. Panel A showed representative images captured on day 4 and 7 following HDACis treatment. Panel B illustrated the encystation ratio, calculated as the number of cells before and after incubation with 0.5% SDS. The line graphs represent the mean ± S.D. from three independent experiments. Statistical differences were determined using repeated measures ANOVA (**P < 0.01 vs. control).

The cyst formation of *N. fowleri* was induced by incubation in MYAS medium for 7 days. The maturation of the cysts was confirmed by their tolerance to 0.5% SDS. Mature cysts were not lysed by 0.5% SDS as shown in Fig. 4A control group. The number of mature cysts of *N. fowleri* gradually increased with incubation time in MYAS medium. From the first day of treatment, the number of mature cysts was significantly reduced by 10 μM of SAHA and JNJ compared to the control (Fig. 4B). SAHA and JNJ exhibited a similar inhibitory effect on the formation of *N. fowleri* cysts. The number of *N. fowleri* cysts showed an insignificant increase until the fourth day of treatment with SAHA and JNJ, after which they decreased to basal levels from the fifth to the seventh day. HDACis, SAHA and JNJ, prevented the trophozoites of *N. fowleri* from transforming into cysts.

### 3.5 SAHA and JNJ enhance the amoebicidal activity of AmB

**Fig. 5:**
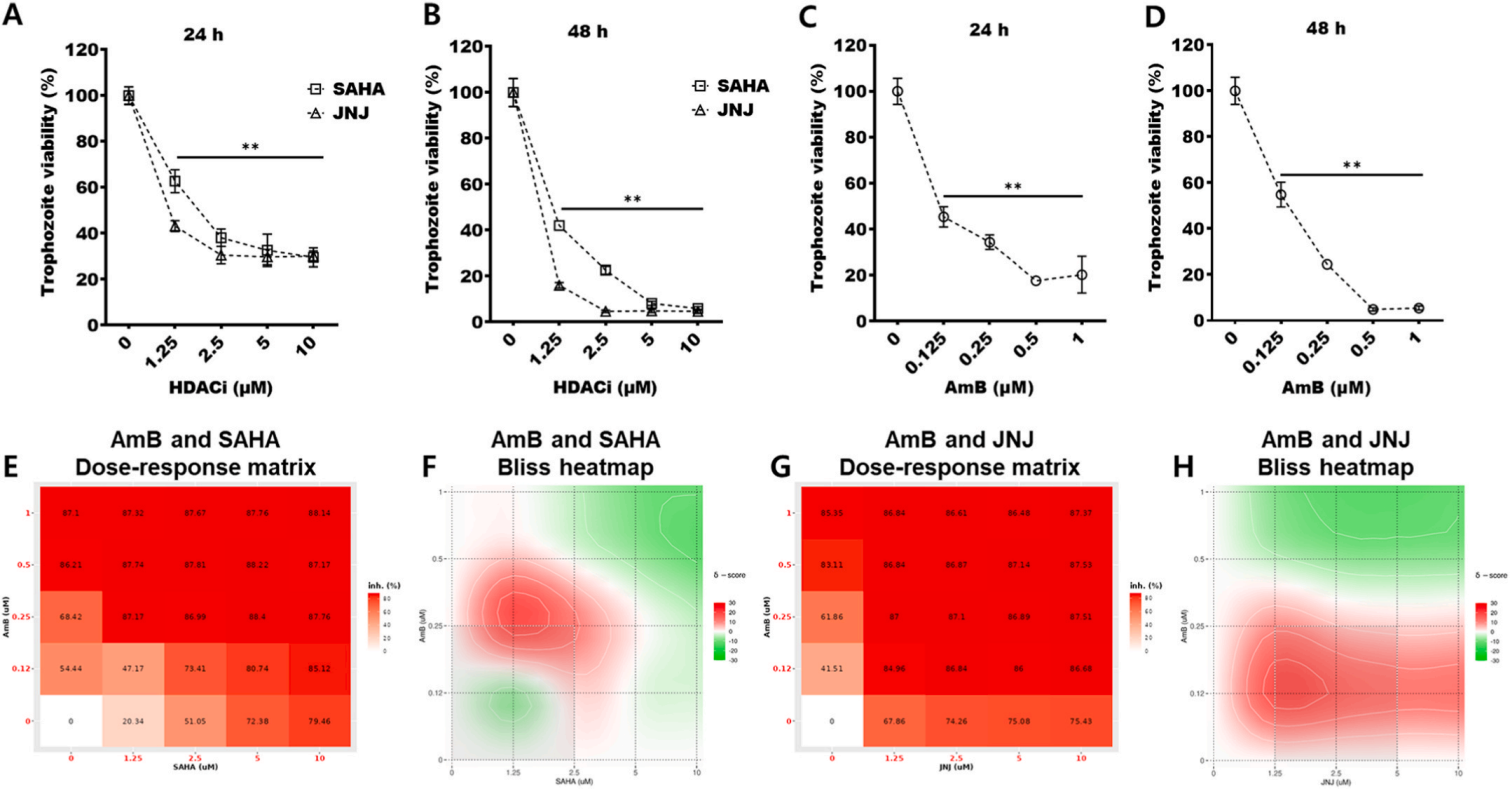
Effects of SAHA, JNJ, and AmB, alone or in combination, on *N. fowleri* viability. Viability was assessed by CCK assay after treatment with serially diluted SAHA or JNJ for 24 h (A) and 48 h (B), or with AmB for 24 h (C) and 48 h (D). The line graphs represent the mean ± S.D. from three independent experiments Statistical significance in A - D was analyzed using Student's t-test (**p < 0.01, control vs. SAHA, JNJ or AmB). Dose–response matrix heatmaps were generated for the combination of AmB and SAHA (E), followed by Bliss/ZIP synergy analyses (F). Using the same approach, dose–response matrix heatmaps (G) and Bliss/ZIP analyses (H) were also performed for the combination of AmB and JNJ. Both HDAC inhibitors exhibited synergistic interactions with AmB at sub-lethal concentrations. At higher concentrations, SAHA showed additive to mildly antagonistic effects, whereas JNJ exhibited an additive effect.

Amoebicidal effects of AmB and its combination with HDACis against *N. fowleri* were evaluated using a Cell counting kit (CCK) assay. The viability of *N. fowleri* decreased in a dose- and time-dependent manner upon single treatment with SAHA, JNJ, and AmB treatment. SAHA and JNJ reduced the viability of *N. fowleri* in a dose-dependent manner. After 24 h of treatment, the IC_50_ values were 2.85 μM for SAHA and 2.23 μM for JNJ (Fig. 5A). Prolonged exposure for 48 h further enhanced their inhibitory effects, with the IC_50_ values decreasing to 1.99 μM and 1.07 μM for SAHA and JNJ, respectively (Fig. 5B). AmB reduced the viability of *N. fowleri* in a concentration- and time-dependent manner. Following 24 h of treatment, the IC_50_ value was 0.22 μM (Fig. 5C), which further decreased to 0.16 μM (Fig. 5D) after 48 h of exposure. To evaluate the combinatorial effects of AmB and HDACis, dose–response matrix heatmaps were generated and synergy was assessed using the Bliss and zero interaction potency (ZIP) models. Bliss analysis of the dose–response matrix for SAHA in combination with AmB (Fig. 5E) suggested synergistic effects at sub-lethal concentrations, whereas predominantly additive or mildly antagonistic interactions were observed at higher concentrations (Fig. 5F). Bliss/ZIP analyses of the dose–response matrix for AmB and JNJ (Fig. 5G) indicated a tendency toward synergistic effects at sub-lethal concentrations, particularly at lower doses of AmB, while interactions at higher concentrations were largely additive (Fig. 5H).

**Fig. 6:**
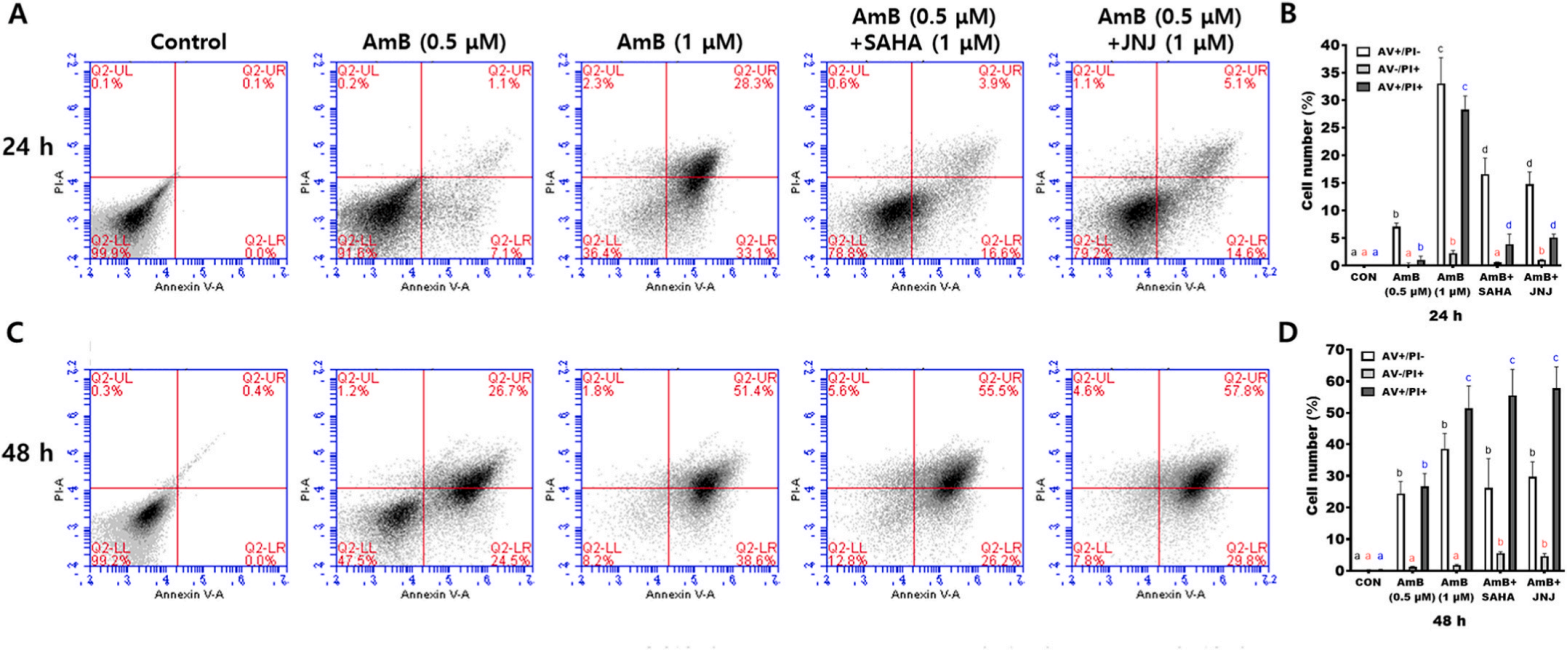
AmB and its combination with HDACis induced apoptosis-like cell death. Apoptosis-like cell death in *N. fowleri* induced by AmB or its combination with HDACis treatment was analyzed using flow cytometry. Single treatment with 0.5 μM AmB produced a time dependent effect, whereas treatment with 1 μM AmB induced drastic apoptosis-like cell death at both 24 and 48 h. To evaluate the combined effects of AmB and HDAC inhibitors, 0.5 μM AmB was co-administered with 1 μM HDACis. Co-treatment with AmB and either SAHA or JNJ resulted in greater levels of apoptosis-like cell death compared with AmB alone for 24 h (A and B) and 48 h (C and D). The bar graphs represent the mean ± S.D. from three independent experiments. Statistical significance was analyzed using one-way ANOVA followed by Dunnett's post hoc test. Different characters indicate statistically significant differences at *p* < 0.05.

**Fig. 7:**
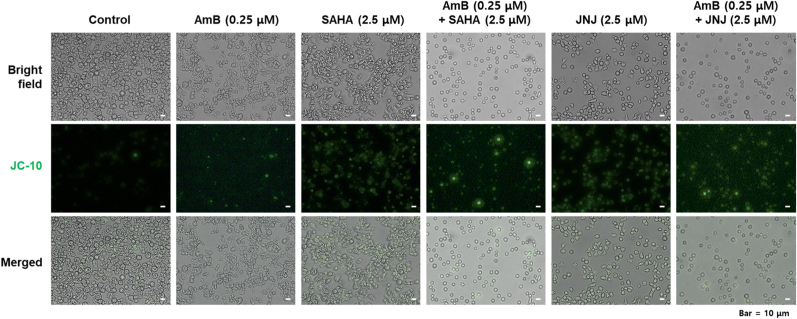
Effect of AmB and HDAC inhibitors on mitochondrial membrane potential. *N. fowleri* cells were treated with AmB, SAHA, and JNJ, either alone or in combination, for 24 h, followed by JC-10 staining for 1 h in the dark. Green fluorescence indicates mitochondrial membrane depolarization.

Apoptosis-like cell death of *N. fowleri* induced by AmB alone or in combination with HDACis was analyzed by flow cytometry. When treated with 0.5 μM AmB for 24 h, *N. fowleri* exhibited 8.2% apoptotic-like cells, including both early and late stages. When combined with 1 μM SAHA or JNJ, the proportion of apoptotic-like cells increased to 20.5% and 19.7%, respectively (Fig. 6A and B). Following treatment of *N. fowleri* with 0.5 μM AmB for 48 h, 51.2% apoptosis-like cell death was observed, whereas combination treatment with SAHA or JNJ increased apoptosis-like cell death to 81.7% and 87.6%, respectively (Fig. 6C and D). At a two-fold higher concentration of AmB (1 μM), 24 and 48 h treatments induced 61.4% and 90% cell death, respectively. To further confirm apoptosis-like cell death in *N. fowleri* at sub-lethal concentrations, mitochondrial membrane depolarization was evaluated. Treatment with AmB (0.25 μM), SAHA (2.5 μM), or JNJ (2.5 μM) alone resulted in modest morphological changes and depolarized mitochondrial membranes in a proportion of cells. In contrast, combination treatment with AmB and HDACis led to more pronounced morphological alterations and a reduction in mitochondrial membrane potential in the majority of cells (Fig. 7).

### 3.6 AmB, SAHA and JNJ showed minimal effect on the viability of human cell line

**Fig. 8:**
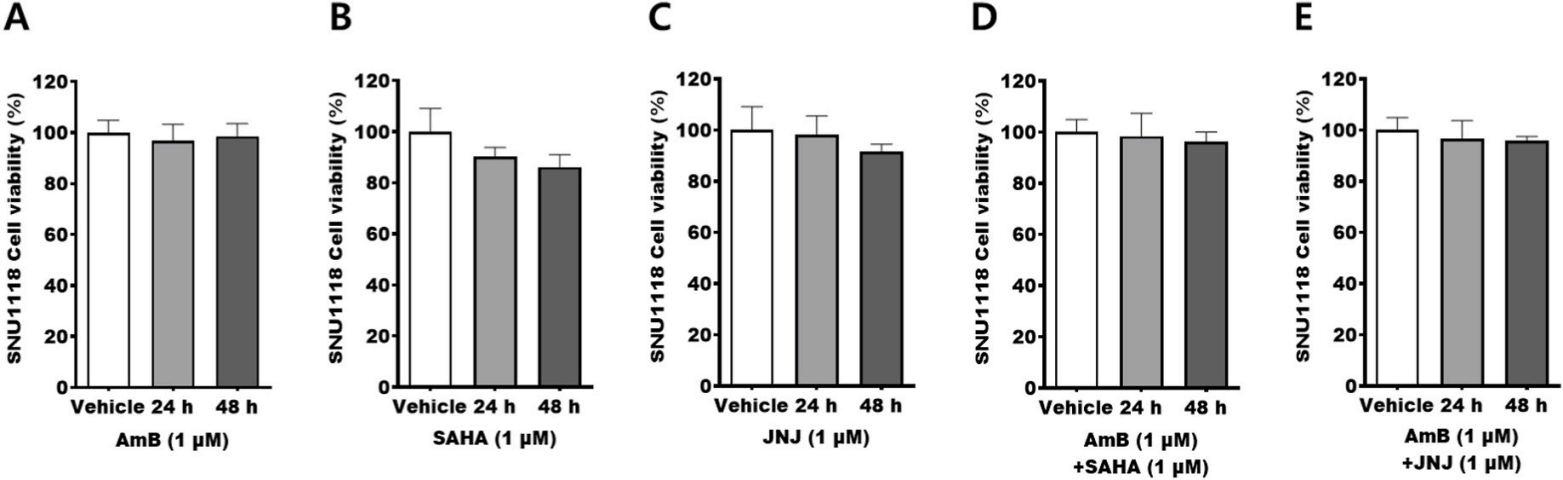
Effect of HDACis on the viability of SNU-1118 cells. The CCK assay was performed after treatment with 1 μM AmB (A), SAHA (B), JNJ (C), or their combinations (D and E) for 24 h and 48 h. No significant change in the viability of SNU-1118 cells was observed. Data are presented as the mean ± S.D. from three independent experiments.

The effects of AmB, SAHA and JNJ on the viability of human brain tumor cell line were investigated using a CCK assay (Fig. 8). SNU-1118 cells were treated with 1 μM AmB (Fig. 8A), SAHA (Fig. 8B), JNJ (Fig. 8C) or their combinations (Fig. 8D and E) for 24 and 48 h. The viability of the SNU-1118 cell line exhibited no statistically significant alterations in relation to the types or combinations of administered agents, nor to the duration of treatment.

## 4 Discussion

The present study aimed to evaluate the effects of various HDACis on the viability and cytotoxicity of *N. fowleri* trophozoites and cysts. The results indicate that the pan-HDAC inhibitors SAHA and JNJ significantly reduced the viability of *N. fowleri* trophozoites, induced cytotoxicity and apoptosis-like cell death, inhibited cyst formation, and enhanced the anti-amoebic activity of AmB, while exhibiting minimal effects on the human cell line.

SAHA is the first HDACi approved by the Food and Drug Administration (FDA) for the treatment of patients with cutaneous T-cell lymphoma (Duvic and Vu, 2007). Previous studies have shown that SAHA exhibits anti-parasitic effects against protozoan parasites such as *Toxoplasma gondii* (Araujo-Silva et al., 2021), *Plasmodium*, and *Trypanosoma* (Engel et al., 2015). The detailed molecular mechanisms underlying the anti-parasitic effects of HDACi are not yet fully understood, although hyperacetylation and gene regulation induced by HDACi treatment in parasites have been previously investigated (Dubois et al., 2009). In this study, the pan HDACis SAHA and JNJ effectively reduced the viability of *N. fowleri* (Fig. 1). The other pan-HDAC inhibitor, JNJ, a second-generation HDACi, has demonstrated more pronounced anti-proliferative effects (Fig. 1B) and induced a substantial increase in apoptosis-like cell death in *N. fowleri* compared to SAHA (Fig. 3A and B). This increased sensitivity of *N. fowleri* to JNJ may be attributed to differences in binding affinity to the target protein and the mechanisms of action between the two pan-HDACis.

Cysts are generally known to contribute to survival under harsh environmental conditions and may be important in the biology of some free-living amoebae. However, in *N. fowleri*, trophozoites are considered the primary pathogenic stage responsible for PAM, and the role of cysts in infection persistence or drug resistance has not been clearly established. Recently, the cysticidal effects of AmB and miltefosine on *N. fowleri* cysts have been reported, with indirect cysticidal activity assessed by incubation with alamarBlue fluorescent dye for 72 h followed by fluorometric measurement (Arberas-Jimenez et al., 2022). In the present study, HDACis were applied during the encystation of *N. fowleri* for 7 days, and treatment with SAHA and JNJ resulted in a marked reduction in the number of mature cysts (Fig. 4). However, this finding should be interpreted with caution, as the current assay does not definitively distinguish direct inhibition of encystation from reduced cyst formation secondary to trophozoite killing. Therefore, although the observed reduction in cyst formation may be of interest, its biological and therapeutic significance in *N. fowleri* remains to be clarified. Further studies, including more refined encystation and excystation assays, will be required to better define the effects of HDAC inhibitors on cyst-related stages.

The LDH assay is a widely used method for assessing cell cytotoxicity, providing valuable insights into the integrity of the cell membrane in response to various treatments. Treatment of *N. fowleri* with SAHA and JNJ showed a concentration- and time-dependent increase in LDH activity (Fig. 2). However, unlike the CCK assay results, where the effect was more enhanced at 48 h compared to 24 h, the LDH activity did not increase further. This is presumably due to the shorter half-life of LDH in the culture medium compared to that in the serum (Riss et al., 2004). Since PI can penetrate damaged cell plasma membranes, nucleic acid staining by PI is typically observed in cells undergoing late apoptosis or necrosis. As shown in Fig. 2, a significant increase in LDH activity was observed 24 h after treating *N. fowleri* with SAHA and JNJ. This increase is presumed to originate partly from the apoptosis-like cell death process and approximately 3% of necrosis, as observed in Fig. 3. It is speculated that the relatively lower cytotoxicity and apoptosis-like effects of HDACis against *N. fowleri*, compared with the viability results, may be due to the fact that the cell-static effects of HDAC inhibitors are not reflected in these assays.

Since its approval by the U.S. FDA in 2006 for the treatment of cutaneous T-cell lymphoma, vorinostat (SAHA) has been associated with various adverse effects, nevertheless, it has continued to be evaluated in clinical trials for multiple cancer types to date (Abdallah, 2026). JNJ, a second-generation pan-HDAC inhibitor that inhibits almost all HDACs in the nanomolar range, is currently undergoing Phase 2 clinical trials for various diseases, including tumors. In clinical trials of JNJ, non-fatal side effects such as fatigue, nausea, decreased appetite, lethargy, and vomiting were reported in Phase 1 trials. Additionally, thrombocytopenia was reported in Phase 2 trials (Venugopal et al., 2013; Child et al., 2016). In vitro cytotoxicity tests with SAHA and JNJ demonstrated that neither SAHA nor JNJ had a significant effect on the viability of SNU-1118, a human brain tumor cell line (Fig. 8). In addition to their relative safety in human cells, another advantage of SAHA and JNJ is that they can be administered orally and can easily cross the blood-brain barrier to reach the brain (Hockly et al., 2003; Lo Cascio et al., 2023). AmB, a drug commonly used to treat granulomatous amoebic encephalitis (GAE) and PAM, is highly effective in its amoebicidal action. However, it has low permeability across the intact BBB (Petraitis et al., 2019), and can cause severe side effects, such as nephrotoxicity. Renal failure resulting from AmB-induced nephrotoxicity may lead to secondary side effects, including electrolyte imbalances such as hypokalemia and hypomagnesemia (Laniado-Laborin and Cabrales-Vargas, 2009). Therefore, to maintain the amoebicidal activity of AmB and reduce its side effects, it is essential to use combination therapy with other drugs, as recommended by the U.S. Centers for Disease Control and Prevention (CDC). To evaluate this concept, we examined the effects of combined treatment with AmB and HDACis. Analysis of the dose–response matrix using the Bliss/ZIP models showed that both SAHA and JNJ exhibited synergistic interactions with AmB at sub-lethal concentrations, while additive effects were observed at higher concentrations (Fig. 5E–H). In addition, co-treatment induced higher levels of apoptosis-like cell death than either AmB or HDACis alone in *N. fowleri* (Fig. 6, Fig. 7). Consistent with previous studies showing that SAHA exhibits no cytotoxicity in normal human cells even at a relatively high concentration of 10 μM (Lee et al., 2020), the present study likewise found no apparent cytotoxicity in human cells following treatment with HDACis alone or in combination with 1 μM AmB (Fig. 8). Together, these results suggest that combining AmB with HDACis may improve therapeutic efficacy while potentially reducing the need for high-dose AmB and its associated toxicity in the treatment of PAM.

In conclusion, this study demonstrated the potential of the pan-HDACis SAHA and JNJ as therapeutic agents against *N. fowleri* infection. SAHA and JNJ inhibited trophozoite viability, induced apoptosis-like cell death, and nearly completely suppressed cyst formation. Notably, because HDACis enhance the anti-amoebic activity of low concentrations of AmB and show no cytotoxicity toward human cells, they offer a promising treatment strategy for PAM that may improve efficacy while reducing AmB-induced side effects.

## CRediT authorship contribution statement

**Hae-Ahm Lee:** Investigation, Methodology, Visualization, Writing – original draft. **Hye-Jeong Jo:** Investigation, Methodology, Visualization, Writing – review & editing. **Ji-Youn Hong:** Formal analysis, Methodology, Validation, Writing – review & editing. **Fu-Shi Quan:** Formal analysis, Resources, Validation, Writing – review & editing. **Eun-Kyung Moon:** Conceptualization, Data curation, Funding acquisition, Investigation, Supervision, Writing – review & editing.

## Declaration of competing interest

The authors declare that they have no known competing financial interests or personal relationships that could have influenced the work reported in this paper.
